# *Mycoplasma pneumoniae* CARDS toxin exploits host cell endosomal acidic pH and vacuolar ATPase proton pump to execute its biological activities

**DOI:** 10.1038/s41598-021-90948-3

**Published:** 2021-06-02

**Authors:** Kumaraguruparan Ramasamy, Sowmya Balasubramanian, Alejandra Kirkpatrick, Daniel Szabo, Lavanya Pandranki, Joel B. Baseman, T. R. Kannan

**Affiliations:** Department of Microbiology, Immunology and Molecular Genetics, University of Texas Health San Antonio, San Antonio, TX 78229 USA

**Keywords:** Microbiology, Pathogenesis

## Abstract

*Mycoplasma pneumoniae* is the leading cause of bacterial community-acquired pneumonia among hospitalized children in the United States. It is also responsible for a spectrum of other respiratory tract disorders and extrapulmonary manifestations in children and adults. The main virulence factor of *M. pneumoniae* is a 591 amino acid multifunctional protein called Community Acquired Respiratory Distress Syndrome (CARDS) toxin. The amino terminal region of CARDS toxin (N-CARDS) retains ADP-ribosylating activity and the carboxy region (C-CARDS) contains the receptor binding and vacuolating activities. After internalization, CARDS toxin is transported in a retrograde manner from endosome through the Golgi complex into the endoplasmic reticulum. However, the mechanisms and criteria by which internalized CARDS toxin is transported and activated to execute its cytotoxic effects remain unknown. In this study, we used full-length CARDS toxin and its mutant and truncated derivatives to analyze how pharmacological drugs that alter pH of intracellular vesicles and electrical potential across vesicular membranes affect translocation of CARDS toxin in mammalian cells. Our results indicate that an acidic environment is essential for CARDS toxin retrograde transport to endoplasmic reticulum. Moreover, retrograde transport facilitates toxin clipping and is required to induce vacuole formation. Additionally, toxin-mediated cell vacuolation is strictly dependent on the function of vacuolar type-ATPase.

## Introduction

*Mycoplasma pneumoniae* is the causative agent of acute and chronic human respiratory diseases and extrapulmonary pathologies^1,2^. *M*. *pneumoniae* infection is the leading cause of bacterial community acquired pneumonia (CAP) among hospitalized children in United States and worldwide^3,4^. It is also responsible for a spectrum of other respiratory tract diseases, including croup, pharyngitis, tracheobronchitis, and bronchiolitis in children and adults^5^. The severity of pulmonary disease caused by *M. pneumoniae* appears to be dependent on biological properties of individual mycoplasma strains and Community Acquired Respiratory Distress Syndrome (CARDS) toxin concentration^6,7^. CARDS toxin is a unique multifunctional protein that exhibits ADP-ribosylating activity like pertussis and diphtheria toxins and vacuolating activity like *Helicobacter pylori* vacuolating cytotoxin^8^. In addition, CARDS toxin is substantially upregulated during *M. pneumoniae* infection of humans and experimental animals^6,7,9–12^. Interestingly, purified recombinant CARDS toxin alone recapitulates many of the respiratory pathologies associated with *M*. *pneumoniae* infection^13^.

Unlike pertussis toxin, CARDS toxin is encoded as a single polypeptide, like diphtheria toxin (DT)^8^. Our three-dimensional structural and functional analyses of CARDS toxin demonstrated the presence of three distinct domains. Domain 1 or N-terminus houses the ADP-ribosylating activity, and domains 2 + 3 or C-terminus are responsible for receptor recognition, binding, internalization, and vacuolization^14,15^. Domain 1 induces interleukin-1β release upon ADP-ribosylation of NLRP3, a major inflammasome protein^16^. Consistent with this observation, we showed that NLRP3 is a critical mediator of hyperinflammation during *M. pneumoniae* infection^17^. Domains 2 + 3 mediate receptor binding and internalization activities and facilitate transport of domain 1 through the cytosol^18^.

Bacterial ADP-ribosylating toxins are synthesized and secreted from their respective pathogen as inactive forms, which are subsequently activated and reach their molecular targets via endosome or endoplasmic reticulum (ER) processing. As in the case of diphtheria toxin, endosome acidification is necessary for cytosolic transfer of its catalytic domain across the endosomal membrane. By contrast, many toxins that follow the retrograde route are transported from endosome to the ER to enter the cytosol. After internalization by clathrin-mediated endocytosis^19^, CARDS toxin is transported in a retrograde manner from endosomes through the Golgi complex to the ER^20^. The retrograde transport of CARDS toxin is mediated by a unique pentapeptide KELED sequence that links the N-terminus ADP-ribosylating domain with the C-terminus vacuolating domain^20^. Prior to cytosol release, activation of many protein toxins occurs by proteolytic clipping at the endosome or ER, often followed by reduction of a disulfide bond. In CARDS toxin, it appears that the disulfide bond formed between cysteines 230 and 247 facilitates proper processing and activation of toxin^21^. Still, how CARDS toxin is properly clipped and activated to exploit host cell machinery and execute cytotoxicity is unclear.

Pharmacological drugs have been widely used to investigate trafficking, translocation and mode of action of bacterial toxins in host cells^22,23^. Using these drugs, intracellular trafficking patterns and translocation mechanisms of DT, Shiga toxin, anthrax toxin, and cholera toxin (CT) have been investigated^22^. Generally, lysosomotropic agents, ionophores and vesicular type proton pump blockers protect host cells against many toxins by neutralizing acidic intracellular pH gradients^23^. However, ammonium chloride (NH_4_Cl), a lysosomotropic amine that blocks DT’s action^24,25^, induces vacuolating activities of *H. pylori* cytotoxin VacA . Earlier, we reported that NH_4_Cl is needed for *Mycoplasma penetrans* toxin MYPE9110 to induce vacuole formation in cultured cervical epithelial cell lines^26^. In addition, we showed that drugs like bafilomycin, which perturbs intracellular acidification, protect host target cells against *M. pneumoniae* CARDS toxin-induced vacuolation^27^. Still, the mechanisms behind this protection have not been elucidated.

In this study, we examined how drugs that alter intracellular vesicle pH affect clipping, trafficking and translocation of *M. pneumoniae* CARDS toxin. Further, the use of full-length (FL) toxin, its mutant and truncated derivatives enabled us to study how acidic pH and, specifically, vesicular electrical potential influence CARDS toxin ER transport, subsequent proper toxin clipping and elicitation of its biological activities.

## Results

### Acidification of endosome is necessary for CARDS toxin-induced vacuole formation

**Figure 1:**
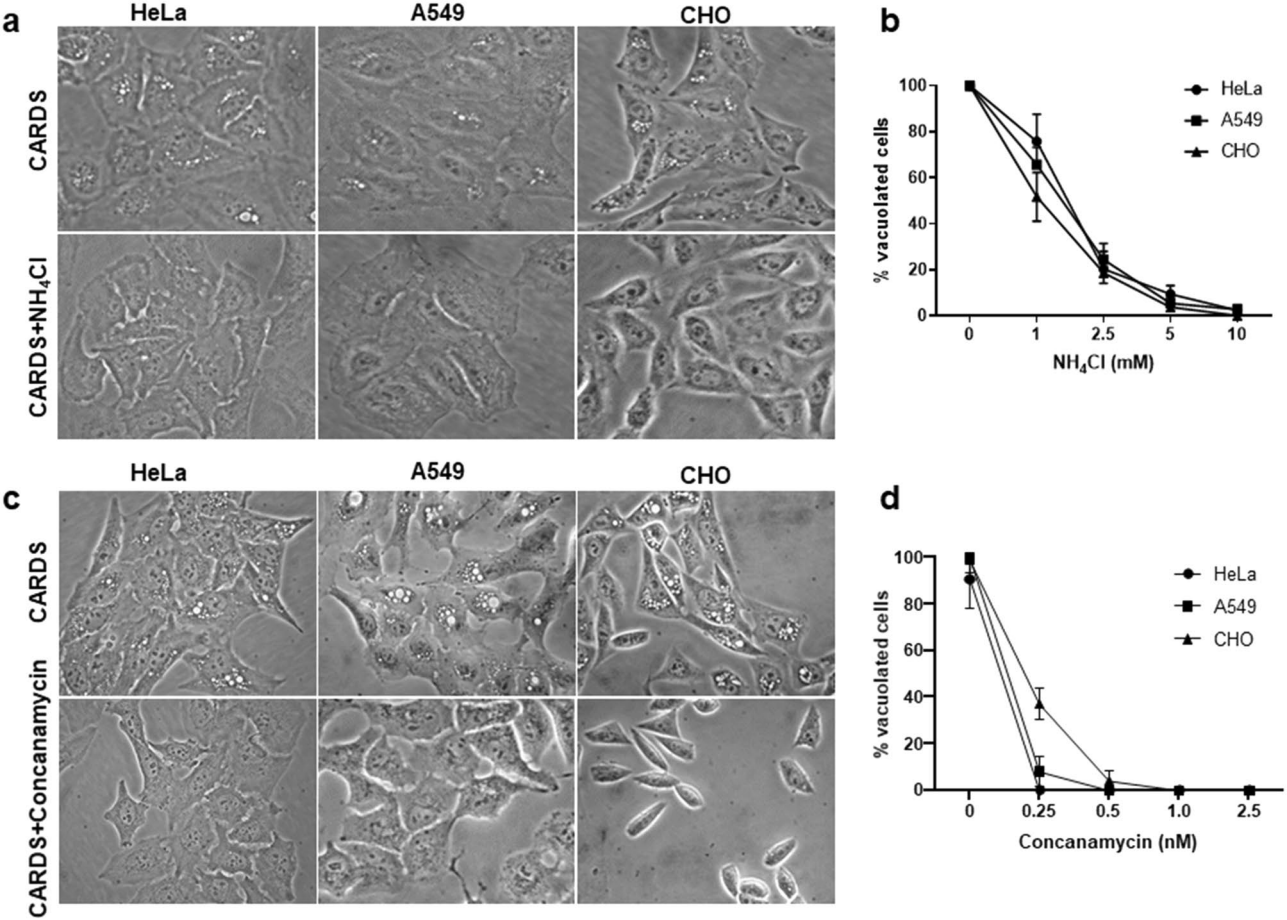
Effect of ammonium chloride (NH_4_Cl) and concanamycin on CARDS toxin-induced vacuoles in A549, HeLa and CHO cells. Mammalian cells pre-treated with or without varying concentrations of NH_4_Cl or concanamycin were incubated with 140 pmol CARDS toxin for 24 h at 37 °C. Images were captured and numbers of vacuolated cells counted. Representative microscopic images of CARDS toxin-treated A549, HeLa and CHO cells in the presence or absence of 5 mM NH_4_Cl (**a**) or 0.5 nM concanamycin (**c**). Numbers of toxin-induced vacuolated cells in the presence of varying concentrations of NH_4_Cl (**b**) and concanamycin (**d**) were counted and expressed as percentage of control cells as indicated in “Methods” section. All values are the mean of two different experiments run in triplicate ± SD.

As NH_4_Cl is known to potentiate vacuole formation of vacuolating cytotoxins in cultured cells^28^, we analyzed its effects on CARDS toxin-induced vacuole formation. NH_4_Cl reduced vacuolation in a concentration (1–10 mM)-dependent manner in human lung carcinoma epithelial (A549) cells (Fig. 1a,b). Also, concanamycin, a compound analogous to bafilomycin that prevents endosomal acidification by selective inhibition of vacuolar ATPase (v-ATPase), inhibited CARDS toxin-induced vacuole formation in a similar dose-dependent manner (0.25–2.5 nM; Fig. 1c,d). In addition, human cervical carcinoma epithelial cells (HeLa) and Chinese hamster ovary (CHO) cells also exhibited concentration-dependent reduction in vacuole formation in the presence of NH_4_Cl, concanamycin and bafilomycin (Figs. 1, S1). These results indicate that, unlike other vacuolating cytotoxins, NH_4_Cl blocks rather than potentiates CARDS toxin-induced vacuole formation.

### Endosomal pH affects CARDS toxin cleavage but not binding and/or entry

**Figure 2:**
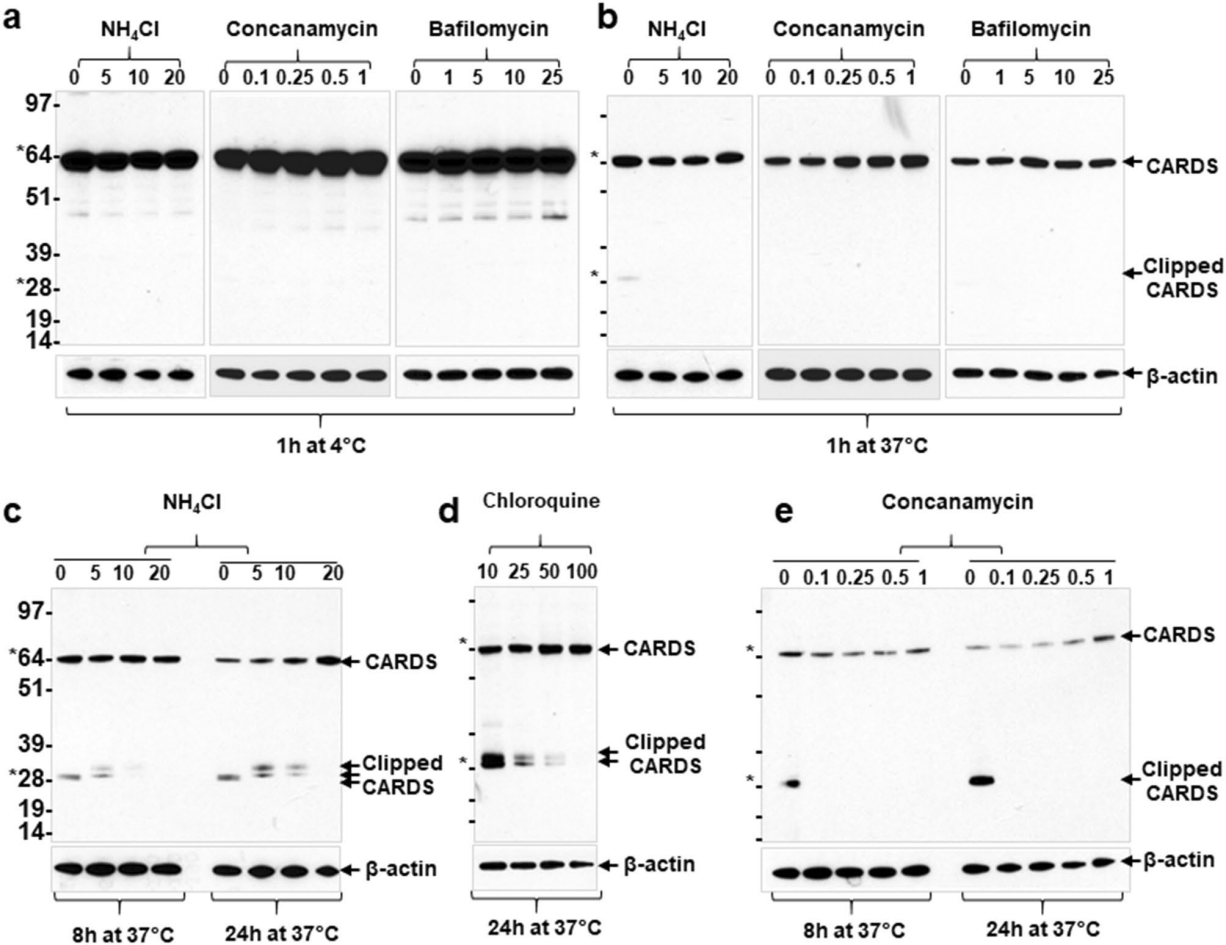
Effect of alkalinizing agents on CARDS toxin binding, internalization and processing. HeLa cells pre-treated with varying concentrations of NH_4_Cl (0–20 mM), concanamycin (0–1 nM) or bafilomycin (0–25 nM) were incubated with 140 pmol CARDS toxin at 4 °C for 1 h. Unbound toxin was removed, and cells were collected and examined for CARDS toxin binding (**a**) or cells were shifted to 37 °C and incubated for 1 h to examine internalized toxin (**b**). Cells treated with CARDS toxin in the presence of NH_4_Cl or concanamycin were further incubated at 37 °C for 8 h and 24 h and analyzed for processed CARDS toxin (**c,e**). Similarly, cells treated with CARDS toxin in the presence of chloroquine and incubated at 37 °C for 24 h were analyzed for FL CARDS toxin and its cleaved products (**d**). Cells from the above conditions were lysed, and CARDS toxin was examined by immunoblot with anti-CARDS polyclonal antibodies. β-actin intensities were used as loading controls. Note that in the absence of pharmacological agents, the band intensity of cleaved CARDS toxin increased in a time-dependent manner. Concanamycin abolished CARDS toxin processing whereas weak bases like NH_4_Cl (5–20 mM) and chloroquine (10–100 ng/ml) resulted in improper clipping of CARDS toxin (**c,e**).

As lysosomotropic agents and v-ATPase blockers are reported to inhibit entry of certain clostridia toxins^29,30^, we analyzed the effect of these compounds on CARDS toxin binding and entry as described in “Methods” section. As shown in Fig. 2, we observed no significant variation in toxin binding (1 h at 4 °C; Fig. 2a) and entry (1 h at 4 °C followed by incubation at 37 °C for 1 h; Fig. 2b) in NH_4_Cl-treated (5–20 mM) or untreated cells. Similarly, we detected no significant difference in binding and entry of CARDS toxin in the presence or absence of concanamycin (0.1–1 nM) or bafilomycin (1–25 nM) (Fig. 2a,b). These results clearly indicate that alkalinizing drugs have no impact on binding and entry of CARDS toxin in mammalian cells.

We also monitored time-dependent processing of CARDS toxin upon internalization and noted its clipping into two fragments (≈ 26 and 28 kDa; Fig. 2c,e). The cleaved CARDS toxin fragments were visible within one hour of internalization, and their intensity increased in a time-dependent manner (1–24 h; Fig. 2b–e). However, the addition of NH_4_Cl (5 mM; the minimal concentration that blocks CARDS toxin-induced vacuole formation) altered the size of clipped toxin fragments (≈ 30 and 36 kDa; Fig. 2c). Further, increased NH_4_Cl concentrations (10 and 20 mM) blocked CARDS toxin cleavage in a dose dependent manner (Fig. 2c). Similarly, chloroquine, a pharmacological drug that acts as a weak base and proton trap, altered the size of clipped CARDS toxin fragments at low concentrations while at high concentrations blocked CARDS toxin cleavage (Fig. 2d). However, at the minimal concentration of concanamycin that blocks vacuole formation, the processing of CARDS toxin was completely abolished (Fig. 2e). Collectively, these results indicate that endosomal acidic pH is critical for CARDS toxin clipping.

### Retrograde transport is also needed for CARDS toxin cleavage

**Figure 3:**
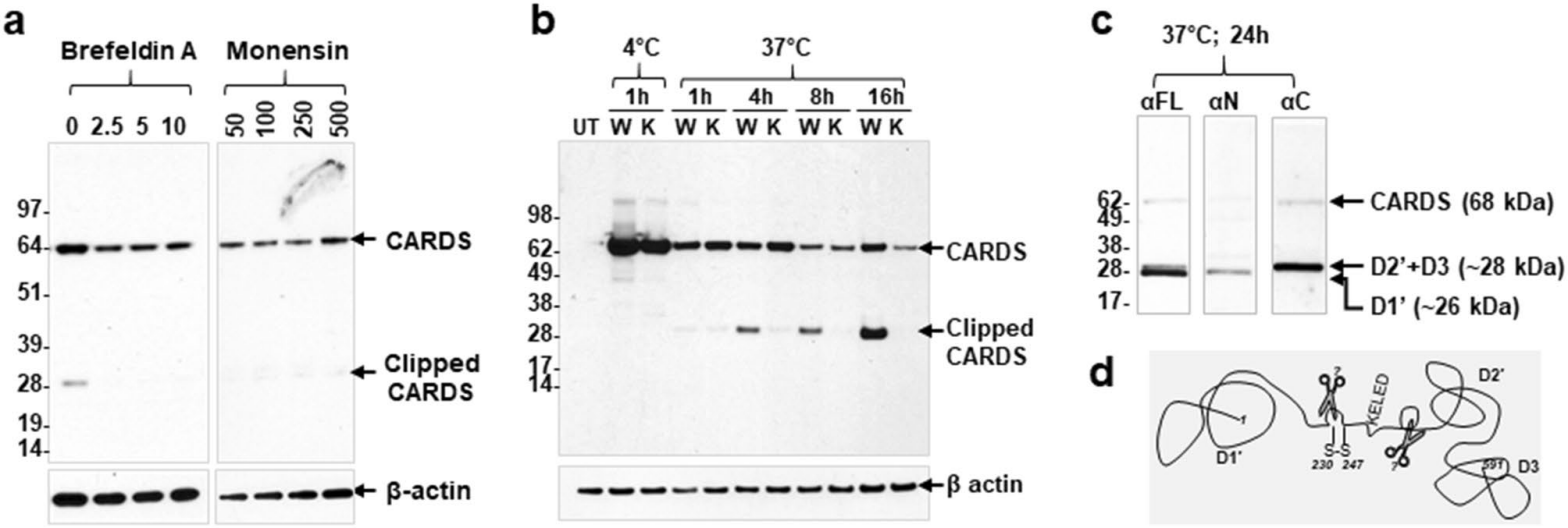
Effect of retrograde transport on CARDS toxin processing. HeLa cells were treated with CARDS toxin or K3-CARDS toxin at 4 °C for 1 h. Unbound toxin was removed, and cells were shifted to 37 °C and incubated up to 24 h. As indicated, cells were lysed at different time intervals and the presence of CARDS toxin was analyzed by immunoblotting with anti-CARDS polyclonal antibodies. Comparative β-actin intensities were used as loading controls. (**a**) Effect of brefeldin A and monensin on CARDS toxin processing. HeLa cells were treated with varying concentrations of brefeldin (0–10 µg/ml) or monensin (50–500 ng/mL) prior to incubating with CARDS toxin for 24 h. Note that in the presence of pharmacological agents, CARDS toxin is not processed. (**b**) Analysis of wild type (W) and K3-mutant (K) CARDS toxin processing. Untreated toxin (UT) HeLa cells served as control. Note that the cleavage of CARDS toxin is readily detected with wild-type toxin but not with K3-mutant. (**c**) Analysis of processed CARDS toxin fragments. CARDS toxin-treated total cell lysates were analyzed at 24 h using anti-CARDS toxin polyclonal antibodies (αFL), anti-N-terminal CARDS toxin antibodies (αN), and anti-C-terminus CARDS toxin antibodies (αC). The same membrane was stripped and probed with the different antibodies. (**d**) Schematic model of CARDS toxin clipping. Disulfide bond between cysteines 230 and 247 is shown as S–S, and the location of the KELED sequence is indicated. Possible protease clipping sites of CARDS toxin within D1 and D2 are indicated as scissors. Note that the depicted schematic of CARDS toxin is a hypothetical illustration and does not represent the structure of CARDS toxin inside the cell. Sizes of molecular weight marker proteins are indicated on the left.

To clarify the relationship between retrograde transport and CARDS toxin cleavage, we compared clipped protein fragments of CARDS toxin in the presence and absence of drugs that block retrograde transport. Monensin, an ionophore known to block transport between the medial and trans cisternae of the Golgi complex^31^ drastically reduced cleavage of CARDS toxin under all tested concentrations (50–500 ng/mL) indicating that cleavage occurs distal to the medial Golgi complex (Fig. 3a). Brefeldin A [2.5–10 µg/ml^32^] treatment also reduced CARDS toxin clipping (Fig. 3a) suggesting that CARDS toxin trafficking to Golgi complex and beyond is required for processing of CARDS toxin.

Previously, we reported that CARDS toxin retrograde transport is mediated by its unique ^268^KELED^272^ motif and that mutation of glutamic acid residues to alanines (K**E**L**E**D to K**A**L**A**D; designated K3-CARDS) markedly reduced CARDS toxin transport to ER^20^. Hence, to further validate the role of retrograde transport in CARDS toxin processing, we compared CARDS- and K3-CARDS-treated cells. Interestingly, in K3-CARDS-treated cell lysates, no processed CARDS toxin was observed even at 24 h, whereas in wild type CARDS toxin-treated cells, cleaved toxin fragments were visible early and increased over time (1–16 h; Fig. 3b). These results further indicate that retrograde transport mediates toxin clipping.

In the absence of pharmacological agents (Figs. 2b–e, 3a) or without blockage of retrograde transport (Fig. 3b, W, 1–16 h), CARDS toxin was processed in a time-dependent manner and yielded two fragments of 26 and 28 kDa sizes. Interestingly, the clipping of CARDS toxin correlated with the time duration of retrograde transport of toxin. To further identify the origin of processed CARDS toxin, we analyzed the fragments using CARDS toxin antibodies raised against different toxin epitopes. Antibody raised against amino acids 1–200, recognized the smaller 26-kDa size fragment that retains the N-terminus-associated ADP-ribosylating activity (Fig. 3c). As indicated in our previous study^21^, CARDS toxin forms the cysteine 230–247 disulfide bond loop within domain1, and like other retrograde toxins, proteolytic cleavage of CARDS toxin within this loop, followed by reduction of the disulfide bond, results in an active ADP-ribosylating domain of approximately 26-kDa (D1’, see schematic Fig. 3d). Similarly, C-terminus antibody raised against CARDS toxin peptide 572–591 recognized a 28-kDa fragment, suggesting that this fragment is composed mostly of domain 2 (D2’, see schematic Fig. 3d) and all of domain 3 (Fig. 3c,d). These results indicate that CARDS toxin is clipped at least in two locations to yield two active fragments (Fig. 3d).

### Deacidification of endosome affects CARDS toxin trafficking

**Figure 4:**
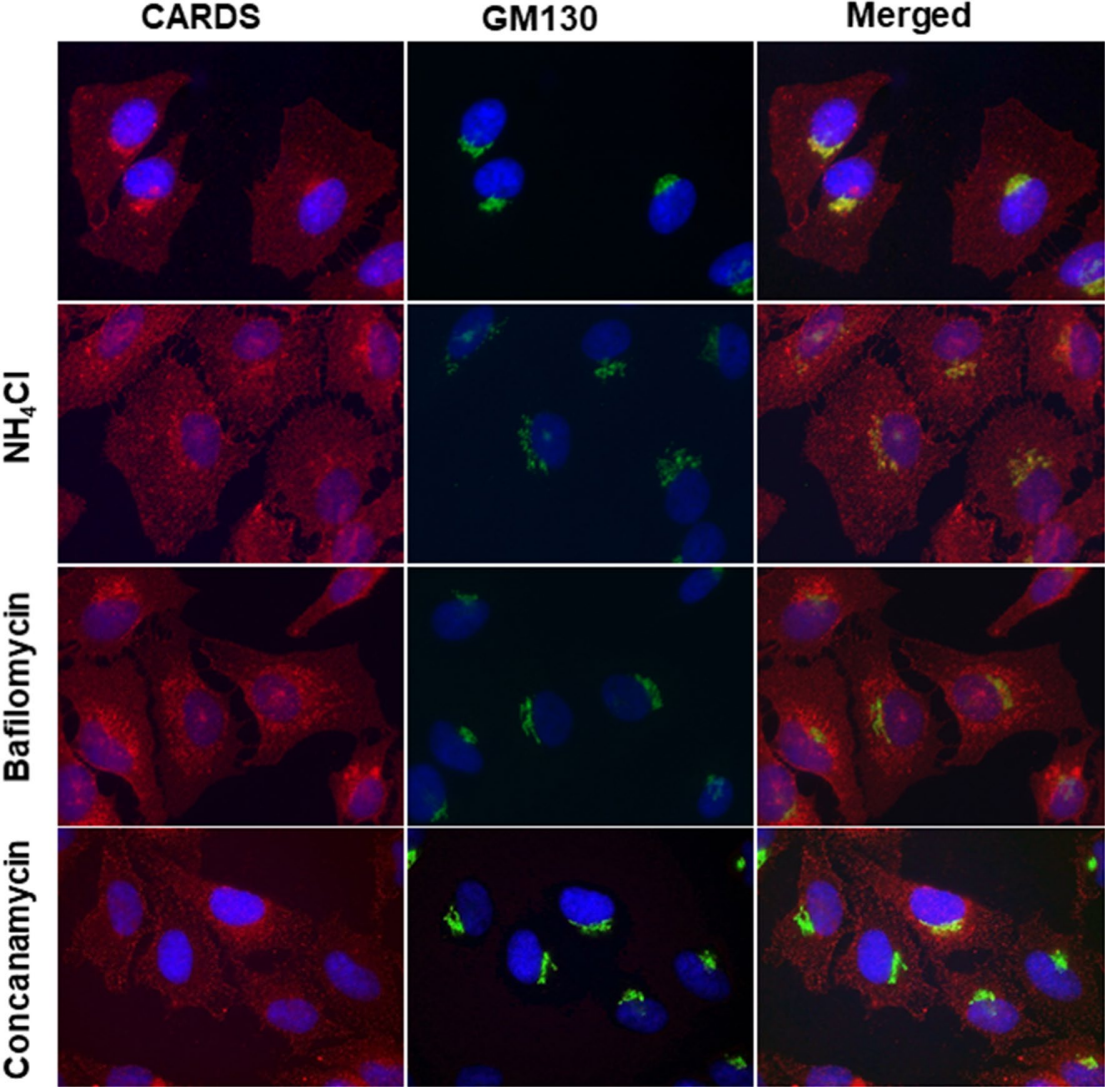
Effect of alkalinizing agents on CARDS toxin transport to Golgi complex. HeLa cells pre-treated with and without NH_4_Cl (5 mM), bafilomycin (10 nM) or concanamycin (1 nM) were incubated with CARDS toxin (140 pmol) at 4 °C for 1 h. Unbound toxin was removed and cells were shifted to 37 °C, incubated for 8 h and fixed in the presence of DAPI to stain nuclei (blue). Fixed cells were probed with rabbit polyclonal anti-CARDS toxin antibodies followed by secondary goat anti-rabbit IgG conjugated with Alexa Fluor 555 (red). To stain Golgi complex, cells were probed with anti-GM130 mouse monoclonal antibodies followed by secondary antibodies conjugated with Alexa Fluor 488 (green). Merged image shows co-localization of CARDS toxin and GM130 as yellow color. Note reduction of CARDS toxin association with GM130 in the presence of pharmacological agents.

To further validate the essential role of acidic endosomal pH on CARDS toxin retrograde transport, we analyzed the trafficking pattern of toxin in the presence or absence of pharmacological agents by co-immunofluorescence (co-IFA). As expected, by 8 h and in the absence of alkalinizing agents, CARDS toxin mostly co-localized with the Golgi complex marker GM130 (Fig. 4, first row merged image). However, in the presence of NH_4_Cl (5 mM), most CARDS toxin distributed throughout the cytoplasm as distinct puncta with a lesser amount co-localized with GM130 (Fig. 4, second row merged image). Thus, dissipation of the pH gradient by NH_4_Cl had a discernible effect on CARDS toxin distribution at the crescent shape structure containing GM130. Similarly, cells treated with bafilomycin (10 nM) or concanamycin (1 nM) showed markedly reduced association of CARDS toxin with the crescent shaped GM130 (Fig. 4, third and fourth rows merged images respectively).

Co-IFA analysis, using endoplasmic reticulum and Golgi intermediate complex (ERGIC) marker ERGIC53, demonstrated reduced co-localization of CARDS toxin with ERGIC in NH_4_Cl (5 mM) or bafilomycin (10 nM)-treated cells. (Supplementary Fig S2). These results indicate that the acidic pH of endosomes plays a vital role in CARDS toxin retrograde transport.

### Endosomal pH alone does not facilitate vacuole formation

**Figure 5:**
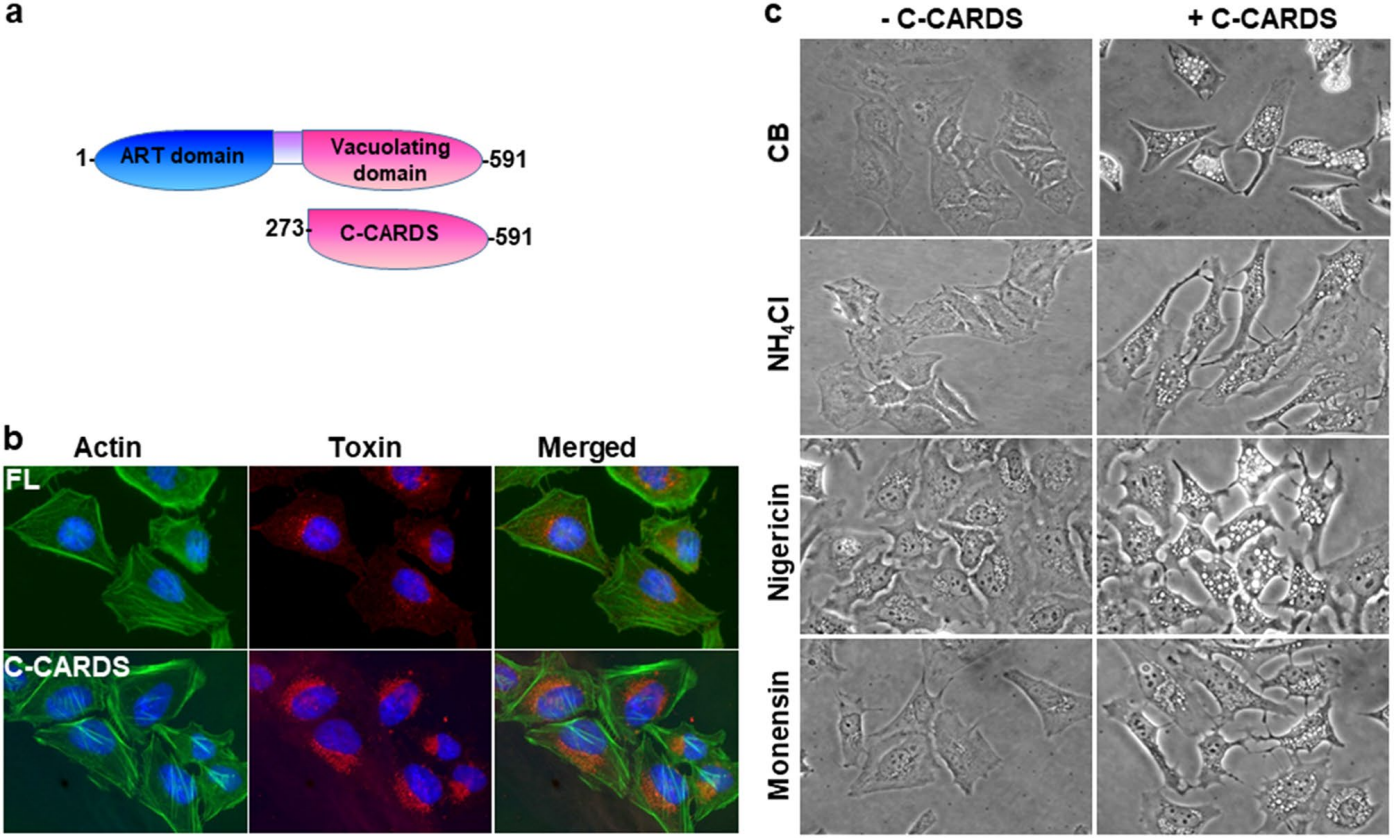
Effect of pH change on clipped CARDS toxin-mediated vacuole formation. (**a**) Schematic representation of C-CARDS used in this study. Carboxy region of CARDS toxin (C-CARDS) was expressed and purified. (**b**) Binding and internalization of full-length toxin (FL) or C-CARDS by HeLa cells. HeLa cells were incubated with FL (140 pmol) or C-CARDS (70 pmol) at 4 °C for 1 h. Unbound toxin was removed, and cells were shifted to 37 °C, incubated for 4 h and fixed in the presence of DAPI to stain nuclei (blue). Fixed cells were probed with Alexa Fluor 488 conjugated phalloidin to stain actin (green) and with rabbit polyclonal anti-CARDS toxin antibodies followed by secondary goat anti-rabbit IgG conjugated with Alexa Fluor 555 (red) to detect FL or C-CARDS. Merged image shows intracellular distribution of FL or C-CARDS. **c**) Effect of pharmacological drugs on C-CARDS-induced vacuoles in HeLa cells. HeLa cells pre-treated with NH_4_Cl (5 mM) or nigericin (25 nM) or monensin (100 ng/ml) were incubated with 70 pmol of C-CARDS (+ C-CARDS) or the carrier buffer (CB; -C-CARDS) for 4 h at 37 °C and images captured.

Our results (Figs. 2, 3, 4) demonstrated that acidic pH is important for CARDS toxin retrograde transport and subsequent clipping, leading to CARDS toxin carboxy region-associated vacuolating activity. However, these results did not clearly distinguish the direct role of acidic endosomal pH on CARDS toxin-induced vacuole formation. To analyze whether pre-clipping of CARDS toxin circumvents the importance of acidic pH in vacuole formation, we generated a deletion construct of CARDS toxin that retains receptor binding and vacuolating properties (i.e., amino acids 273–591, designated C-CARDS; schematic Fig. 5a).

To characterize the binding and internalization of C-CARDS, we performed IFA and, as expected, C-CARDS bound to and was internalized by target cells (Fig. 5b). However, unlike FL toxin, pre-incubation of cells with NH_4_Cl, did not prevent C-CARDS-induced vacuoles (Fig. 5c). Similarly, ionophore nigericin, which abolishes acidification by exchanging luminal protons (H^+^) for cytosolic monovalent cations^23^, had no effect on C-CARDS-induced vacuolation (Fig. 5c). In contrast, nigericin inhibited vacuole formation of FL toxin (data not shown). In addition, the presence of ionophore monensin (100 ng/mL), unlike FL toxin^27^, had no effect on C-CARDS toxin-induced cytotoxicity (Fig. 5c). The differential sensitivity of FL toxin versus C-CARDS towards these drugs implies that the acidic endosomal pH is essential for FL toxin clipping and release of active C-CARDS domain, leading to vacuolating activity.

### V-ATPase-mediated membrane potential is essential for CARDS toxin-induced vacuole formation

**Figure 6:**
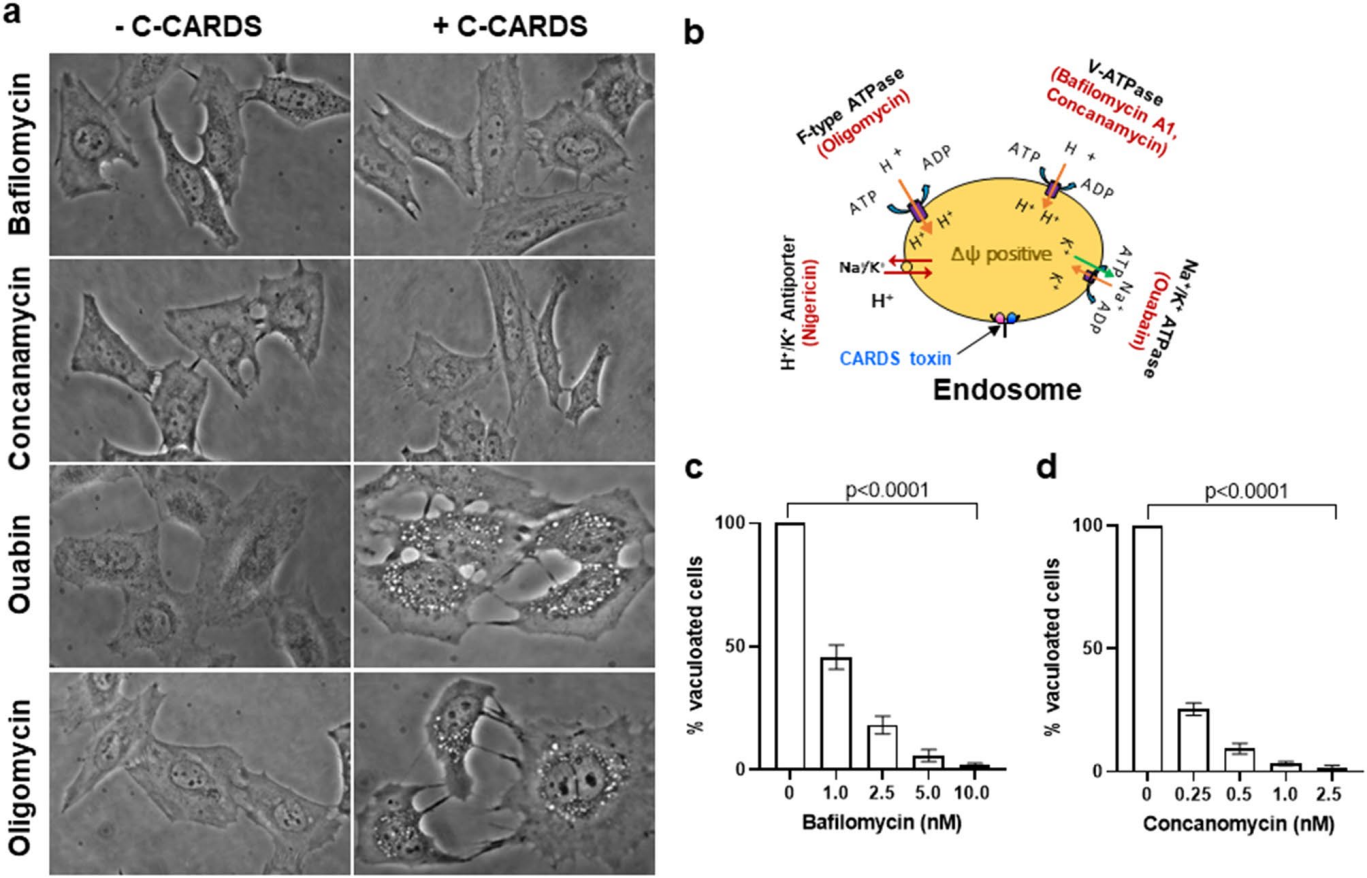
Effect of ATPase blockers on C-CARDS-mediated vacuole formation. Bafilomycin (10 nM), concanamycin (1 nM), ouabin (100 nM) and oligomycin (100 ng/mL) were compared for their impact on C-CARDS-induced vacuoles in HeLa cell lines. Mammalian cells pre-treated with ATPase blockers were incubated with 70 pmol of C-CARDS or carrier solution for 3 h at 37 °C. Images were captured and numbers of vacuolated cells quantified. (**a**) Representative microscopic images of each ATPase blocker-treated cells in the presence or absence of C-CARDS. (**b**) Schematic representation of action of ATPase blockers used in this study. (**c,d**) Numbers of toxin-induced vacuolated cells in the presence of ATPase blockers bafilomycin (**c**) and concanamycin (**d**) were counted and expressed as percentage of control cells. All values are the mean of two different experiments run in triplicate ± SD.

In contrast to the lysosomotropic amines and ionophore drugs, the v-ATPase blockers bafilomycin and concanamycin completely inhibited C-CARDS toxin-induced vacuolation (Fig. 6a,d). Other than v-ATPase, Na^+^/K^+^-ATPase and F-type ATPase are important for generating an electrochemical gradient by pumping ions and protons into the vesicles, respectively (Fig. 6b). To elucidate the role of Na^+^/K^+^-ATPase and F_1_F_0_-type ATPase, we used their inhibitors ouabin (50–250 nM) or oligomycin (50–250 ng/mL) respectively. These ATPase blockers did not alter C-CARDS toxin-induced vacuole formation in host cells (Fig. 6a) indicating that the electrical potential across the vesicle membrane generated by V-type proton pumps is important for CARDS toxin-induced vacuolation.

## Discussion

Certain viruses and toxins execute their biological activities by exploiting the acidic environment of the host cell^23,33^. For example, specific endocytosed toxins, such as DT, translocate out of the endosome in a manner that requires low pH^34,35^. In contrast, CT’s catalytic chain translocates into the cytosol from the ER after retrograde transport^36^. Although CARDS toxin utilizes the vesicular retrograde pathway to reach the ER and induce vacuole formation^20^ the latter is blocked by bafilomycin^27^ indicating that CARDS toxin needs an acidic environment to exert its activity. To further determine the role of endosomal pH in CARDS toxin activation, we used specific pharmacological drugs to uncover the mechanism(s) by which CARDS toxin is activated.

Potentiation of vacuolating activity of bacterial toxins by NH_4_Cl has been reported for cytotoxins VacA from *H*. *pylori*^28^ and MYPE9110 from *M*. *penetrans*^26^. In contrast, quenching the pH of intracellular compartments by weak bases NH_4_Cl and chloroquine or by v-ATPase blockers bafilomycin and concanamycin, blocks CARDS toxin-induced vacuolation indicating that CARDS toxin uses an alternative pathway for activation (Figs. 1, 2). Interestingly, previous studies have demonstrated that lysosomotropic amines NH_4_Cl or chloroquine completely protect cells from DT^25^ and anthrax toxin^37^. However, these toxins follow a shorter route (endosome to cytosol) than CARDS toxin’s complicated retrograde pathway (endosome → Golgi complex → ER to cytosol) to execute cytotoxicity^20^.

Many bacterial and plant toxins undergo proteolytic processing by host cell proteases^38,39^ which is necessary for the subsequent translocation and release of the catalytic domain. In the present study, once CARDS toxin translocated from endosomes, it was eventually cleaved by unknown host protease(s) into at least two stable products (≈26 kDa and 28 kDa peptides) (Fig. 2). Interestingly, the pH change induced by weak bases NH_4_Cl or chloroquine or by v-ATPase blockers bafilomycin and concanamycin altered the size of the processed CARDS toxin peptides or prevented CARDS toxin clipping, respectively, thereby, affecting subsequent C-CARDS-induced vacuole formation. Regardless of the absence of distinct cleaved peptides, the FL toxin was not stably maintained over time in the presence of the V-ATPase blockers. In addition, the differential proteolysis of CARDS toxin in the presence of weak bases suggests a role for multiple host proteases or partial processing or alterations in toxin secondary structure. Further studies are warranted to address the mechanisms by which CARDS toxin is processed (Fig. 2). Similarly, compounds that block retrograde transport, such as monensin and brefeldin A, abolished CARDS toxin cleavage demonstrate that toxin trafficking from endosomes to ER is essential for toxin processing (Fig. 3a). Also, CARDS- KELED retrograde-deficient mutant clearly revealed no toxin processing, indicating that ER transport is important for CARDS toxin processing (Fig. 3b). Upon retrograde transport, FL CARDS toxin is clipped into ~ 26-kDa N-terminus and ~ 28-kDa C-terminus fragments. As indicated in our previous study^21^, CARDS toxin forms a disulfide bond between two cysteine residues at positions 230–247, and the loop formed between these two residues is sensitive to host protease(s). The released 26-kDa N-terminus fragment indicates proteolytic cleavage of CARDS toxin within this loop, followed by reduction of the disulfide bond, and results in an active ADP-ribosylating domain of approximately 26-kDa (Fig. 3c&d); this mechanism of activation is observed in other retrograde toxins. Similarly, the clipped 28-kDa C-terminus fragment suggests that this fragment is devoid of ER retention signal sequence KELED and composed mostly of domain 2 and all of domain 3 (D2’ + D3; Fig. 3c,d).

**Figure 7:**
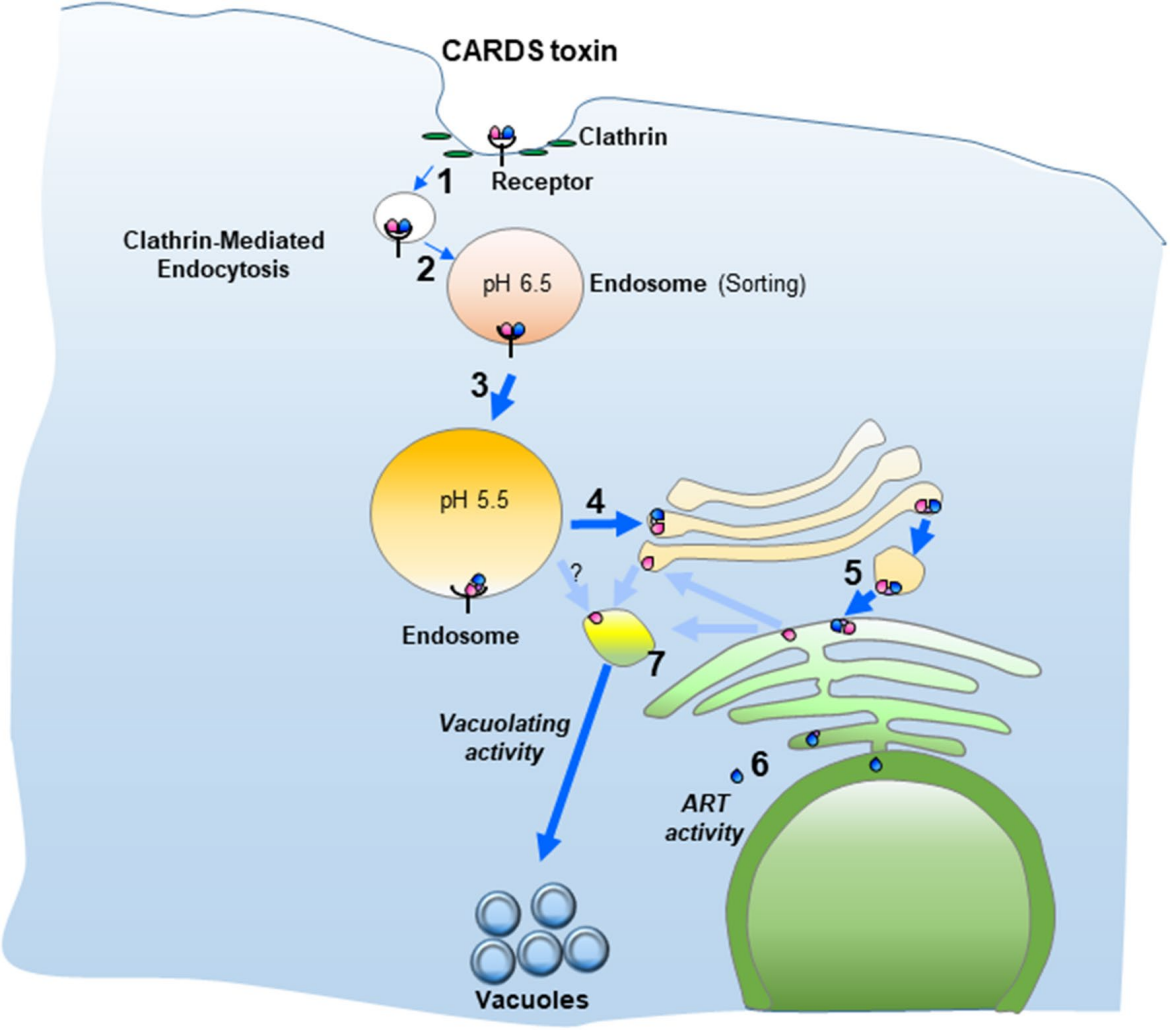
Illustrative model for CARDS toxin-mediated cytotoxicity. CARDS toxin is internalized via clathrin-mediated endocytosis (steps 1–3). Acidic environment of the endosome enables retrograde translocation of CARDS toxin to Golgi complex and ER (steps 4–5). Clipping of CARDS toxin during its retrograde transport by unknown host protease(s) facilitates release of active N-terminus (blue oval) to elicit ADP-ribosyl transferase (ART) activity and C-terminus (pink oval) to induce vacuole formation (steps 6 & 7).

As we reported previously with the KELED mutant^20^, specific pharmacological compounds also prevented subsequent vacuole formation (Figs. 2, 3). Hence, it is possible that neutralization of the acidic endosomal pH trapped CARDS toxin within endosomal vesicles, as observed with DT and other toxins that follow the pH-dependent retrograde pathway, reinforcing the role of the acidic environment in altering toxin conformation and facilitating translocation^40^ from endosome to ER to enter the cytosol. Indeed, v-ATPase blockers inhibited time-dependent localization of internalized CARDS toxin at the Golgi complex without substantially affecting the initial internalization steps (Fig. 4). However, a less efficient inhibition of Golgi complex colocalization of CARDS toxin was observed in the presence of NH_4_Cl (Fig. 4). The difference in the inhibitory effects on these two types of compounds suggests that deacidification of endosomes is less pronounced in the presence of the weak base NH_4_Cl than the v-ATPase blockers bafilomycin and concanamycin. Interestingly, in CARDS toxin, besides serving as sites of cleavage, N- and C- termini possess the ER retention sequence linker KELED necessary for transport of CARDS toxin to the ER. Thus, upon clipping and disulfide bond reduction, the ADP-ribosylating N-terminal domain might be released into the cytosol or other organelles, and the clipped carboxy-terminal vacuolating domain might be shuttled back through the Golgi network to reach the endosome in a possible Rab9-dependent manner^27^ to induce vacuole formation (Figs. 3, 7). Altogether, CARDS toxin cleavage is complex and essential for intoxication and vacuolation. Furthermore, retrograde transport of toxin precedes processing and eventual release of active domains.

To further distinguish the role of endosomal acidic pH in CARDS toxin processing and vacuole formation, we used C-CARDS, a pre-clipped CARDS toxin domain that houses vacuolating activity, along with FL toxin, and compared their vacuolating activities in the presence of alkalinizing agents. The distinct differences in the sensitivity of FL and C-CARDS toxins towards these drugs (Fig. 1, 5) suggest that dissipation of the pH gradient across the vesicle membrane does not directly block vacuolation associated with CARDS toxin. Further, unlike the Na^+^/K^+^-ATPase blocker (ouabin) and F_1_F_0_-ATPase blocker (oligomycin A), v-ATPase blockers bafilomycin and concanamycin arrested both FL and C-CARDS induced vacuole formation (Figs. 1, S1. 6). Therefore, the inside-positive electrical potential across the vesicle membrane, generated by V-type proton pumps, may be required for CARDS toxin-induced vacuolation.

Based on our prior published studies and the results derived here, we offer a model for CARDS toxin-mediated cytotoxicity (Fig. 7). To summarize, upon binding to host cell receptors, CARDS toxin is internalized by clathrin-mediated endocytosis. Following internalization, a substantial amount of toxin is routed to the lysosomes for degradation. The remaining toxin is transported through acidic endosome compartments and subsequent routing to the Golgi complex. An additional vesicle trafficking step delivers the toxin to the ER. Intact toxin may shuttle between the Golgi complex and ER until it is reductively cleaved to separate the ADP-ribosylating domain (blue oval) away from the receptor binding and vacuolating domain (pink oval). The clipped N-terminus elicits ADP-ribosyl transferase activity whereas the C-terminus translocates from ER to the Golgi complex, ultimately inducing vacuole formation through unknown mechanisms. Based on the above results, we conclude that CARDS toxin trafficking from endosomes to ER is essential for toxin-mediated vacuole formation. Additionally, an acidic environment is necessary to mediate toxin’s retrograde transport and subsequent processing so that the active N-terminus and C-terminus domains are released to execute their biological activities. Further, cell vacuolation induced by CARDS toxin is strictly dependent on the function of vacuolar type-ATPase.

## Methods

### Mammalian cell cultures and growth

Human cervical adenocarcinoma HeLa cells (CCL-2), human alveolar adenocarcinoma A549 cells (CCL-185), and Chinese Hamster ovary CHO-K1 cells (CCL-61) were obtained from the American Type Culture Collection and cultured at 37 °C in 95% air − 5% CO_2_ in minimal essential medium (MEM) or Kaighn’s modification of Ham’s F-12 medium (F12K) supplemented with 10% fetal bovine serum (HyClone), 2 mM L-glutamine, 1,000 U/ml penicillin G, and 50 µg/ml streptomycin (Invitrogen).

### Bacterial strains, plasmids, and DNA manipulations

The following vectors and bacterial cells were used to generate truncated CARDS toxin and its derivatives: pCR2.1 (TA cloning vector; Invitrogen) and *Escherichia coli* INVαF' (F' endA1 rec1 hsdR17 supE44 gyrA96 lacZΔM15 [lacZYA^-^argF]) for gene manipulations and pET-19b (N-terminal His10 tag, expression vector; Novagen/EMD Biosciences, San Diego, CA) and lipid A-deficient *E. coli* BL21(DE3) (lpxM F^−^ ompT hsdSB [rB^−^ mB^−^] gal dcm) for protein expression.

### Cloning, expression and purification of recombinant proteins

CARDS toxin and its derivatives were purified as described before^8^. In brief, plasmids encoding FL and K3-CARDS toxin mutant were transformed into *E. coli* BL21, induced with 1 mM isopropyl-β-d-1-thiogalactopyranoside (IPTG) at 18 °C for 16 h and purified as histidine-tagged proteins according to the manufacturer’s instructions (GE Healthcare). C-CARDS (273–591) encoding DNA fragment was amplified using the forward primer 5′‐CATATGACACCAGTATACCTA AGGGGAATTAAAACG‐3′ and the reverse primer 5′‐ CGTTAAAGGATCCTCGCTAAAAGC GATC‐3′, which produces NdeI and BamHI (underlined) sites at the 5′‐ and 3′‐ends of the amplified DNA fragment, respectively; ligated into pCR2.1 vector; and then sub-cloned in pET19b (pET-C-CARDS). Subsequently, the plasmid  was transformed in *E. coli* BL21 and overexpressed by induction with 1 mM IPTG at 25 °C for 16 h. The soluble recombinant fusion protein was purified by nickel-nitrilotriacetic acid (Ni–NTA) affinity chromatography under native conditions (GE health care). Protein-containing fractions were pooled, desalted in 50 mM Tris–HCl buffer (pH 7.4) and 5% glycerol by using PD-10 columns and stored at − 80 °C in aliquots.

### Pharmacological drugs treatment

Pharmacological agents bafilomycin, concanamycin, ammonium chloride (NH_4_Cl), brefeldin A, monensin, chloroquine, ouabin, oligomycin and nigericin were purchased from Sigma. HeLa, A549 and CHO cells were pre-treated with different concentrations of bafilomycin (1–25 nM), concanamycin (0.1–10 nM) and NH_4_Cl (1–20 mM) in complete medium for 1 h at 37 °C before addition of CARDS toxin (140 pmol). In addition, HeLa cells were treated with monensin (50–500 ng/ mL), chloroquine (10–100 ng/mL), brefeldin A (2.5–10 µg/ml), ouabin (50–250 nM), oligomycin (50–250 ng/mL) and nigericin (5–100 nM) prior to the addition of optimized concentrations of FL, K3-CARDS toxin (140 pmol) or C-CARDS toxin (70 pmol). Cell vacuolation was recorded and quantified as described in the vacuolation section. For western blot analysis, HeLa cells were grown in 6-well plates and treated with specific pharmacological inhibitors as indicated above. At specific time points, cells were harvested, lysed with NP-40 lysis buffer and cell lysates were stored in aliquots at − 80 °C. For immunofluorescence analysis (IFA), HeLa cells were seeded on cover slips in 24-well plates and pre-treated with or without NH_4_Cl (5 mM), bafilomycin (10 nM) and concanamycin (1 nM) in complete medium for 1 h at 37 °C. After pre-treatment, CARDS toxin (140 pmol) was added to cells in cold serum-free medium, and plates were transferred to 4 °C for 1 h. Unbound CARDS toxin was removed by washing with cold serum-free medium, and pre-warmed complete medium along with pharmacological agents was added. At indicated time intervals (1–8 h), cells were fixed and analyzed for binding and internalization of CARDS toxin as described in the IFA section.

### Mammalian cell vacuolation

Cells seeded in 6-well plates and grown to 60% confluency were treated with toxin and its derivatives in the presence and absence of pharmacological agents and analyzed for vacuole formation as described earlier^18^. In brief, cells were examined microscopically for vacuole formation, and images were recorded at regular intervals and vacuoles quantified. The timing of vacuolization, number of vacuoles per cell, size of the vacuoles, and number of vacuolated cells were observed at different time points. All experiments were repeated in triplicate, and 20 fields of 20 to 25 cells per sample were examined to determine vacuolization patterns. Statistical analysis was performed using Graph pad prism.

### Western blot

HeLa cells grown in 6-well plates were treated with CARDS toxin in the presence and absence of pharmacological agents as mentioned above. Briefly, medium was removed from culture plates followed by two washes with ice-cold PBS. Subsequently, cells were collected by scraping, lysed with NP-40 lysis buffer and kept on ice for 30 min. For immunoblotting, total cell lysates were denatured in sample lysis buffer, subjected to Nu-PAGE on 4–12% gel (Invitrogen), and separated proteins were transferred onto 0.2 µm nitrocellulose membranes (Bio-Rad) using semi dry transfer (Bio-Rad). After treating membranes with blocking buffer (5% non-fat dry milk in Tris-buffered saline buffer pH 7.4 with 0.1% Tween 20) for 1 h at RT, membranes were probed with rabbit polyclonal anti-CARDS toxin antibodies (1:1,000 dilution) raised against full length (1–591; recognizes both N- and C-terminus of CARDS toxin) and N-terminus (1–200) and C-terminus (572–591) peptides over night at 4 °C followed by peroxidase-conjugated secondary anti-rabbit antibody (1:5,000 dilution; Invitrogen) and visualized by ECL detection system (Thermo Fisher Scientific). Each membrane was stripped and re-probed with specific CARDS toxin antibodies or mouse anti-β actin antibody (1:10,000 dilution; Sigma) followed by anti-mouse secondary antibody (1:10,000 dilution, Invitrogen), to ensure equal protein loading.

### Immunofluorescence microscopy

Immunofluorescence analysis (IFA) was performed as described previously^20^. Briefly, HeLa cells (2 × 10^4^ cells/well) grown on glass coverslips treated with or without specific pharmacological agents and CARDS toxin (140 pmol) or C-CARDS (70 pmol) were fixed in 2% paraformaldehyde, permeabilized with 0.1% Triton-X-100 and blocked with 1% normal goat serum (NGS; Gibco). Then, cells were washed with 0.2% NGS and treated with rabbit polyclonal anti-CARDS toxin (1:1000) as indicated previously and incubated with secondary goat polyclonal anti-rabbit antibody (1:1000 dilution) labeled with Alexa Fluor 555 (Invitrogen) for 1 h. Cellular F-Actin was stained with Alexa Fluor 488-conjugated phalloidin (Invitrogen). For co-IFA studies, cells were incubated with GM130 (1:1000, Abcam) or ERGIC (1:100; Santa Cruz) monoclonal antibodies in PBS with 0.2% NGS in PBS for 1 h. Cells were washed with PBS containing 0.2% NGS and incubated with secondary antibodies (Alexa Fluor 488 goat anti-mouse, 1:500) in PBS with 0.2% NGS for 1 h at RT. Individual samples were mounted in medium containing 4’,6-diamidino-2-phenylindole (DAPI; Vector Laboratories;), and images were acquired using a Carl-Zeiss immunofluorescence microscope, and Z sections were prepared using AxioVision deconvolution software and enhanced in Adobe Photoshop.

### Statistical analysis and reproducibility of experiments

All experiments were performed at least three independent times, and representative figures were shown in the Results section. All data are expressed as the mean ± standard error of the mean of triplicates. All statistical analyses were performed using GraphPad Prism 8 software (GraphPad Software, San Diego, CA). *P* value less than 0.05 was considered significant.

## Supplementary Information

Supplementary Information.
