# Supplementary material for: Mycoplasma pneumoniae CARDS toxin exploits host cell endosomal acidic pH and vacuolar ATPase proton pump to execute its biological activities
Source: Sci Rep. 2021 Jun 2;11:11571. doi: 10.1038/s41598-021-90948-3 (PMC8172646; doi:10.1038/s41598-021-90948-3)
Supplement: Supplementary file 1 — Supplementary Information. [file 41598_2021_90948_MOESM1_ESM.pdf]

Mycoplasma pneumoniae CARDS toxin exploits host cell endosomal acidic pH and vacuolar ATPase proton pump to execute its biological activities

Kumaraguruparan Ramasamy, Sowmya Balasubramanian, Alejandra Kirkpatrick, Daniel Szabo, Lavanya Pandranki, Joel B. Baseman and T. R. Kannan*

Supplementary Figures

Figure S1

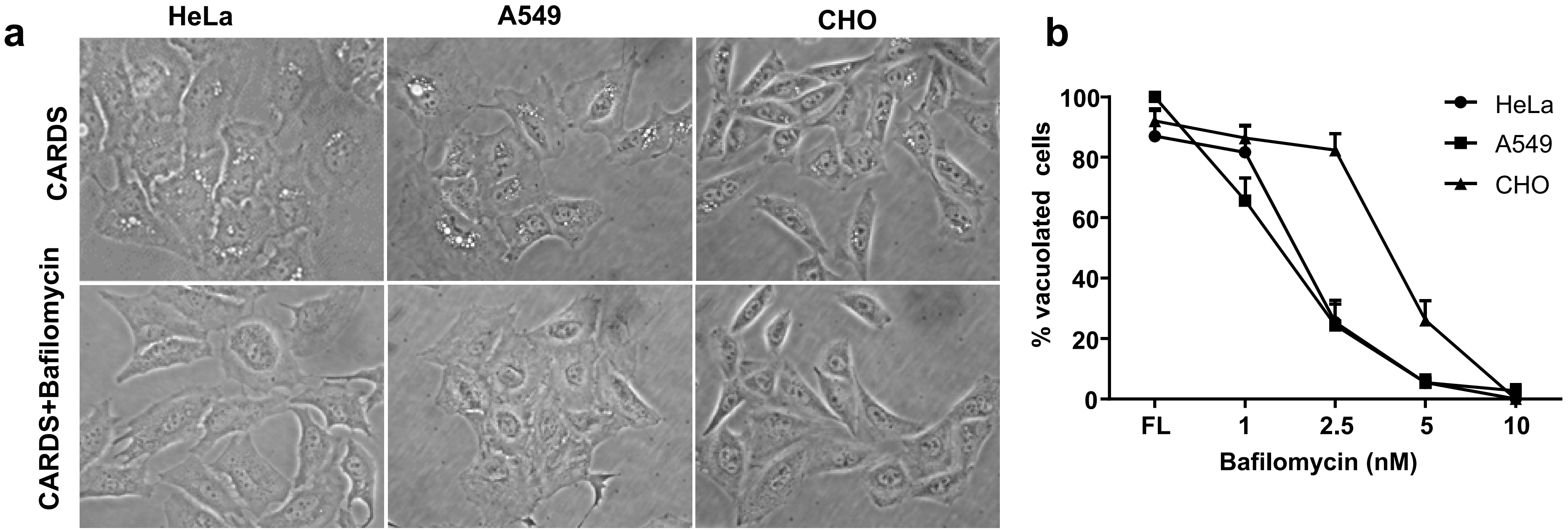

Figure S1. Effect of bafilomycin on CARDS toxin-induced vacuoles in A549, HeLa and CHO cell lines. Mammalian cells pretreated with or without varying concentrations of bafilomycin were incubated with 140 pmol CARDS toxin for 24 h at 37°C and images captured and numbers of vacuolated cells counted. **a**) Representative microscopic images of CARDS toxin treated A549, HeLa and CHO cell lines (24 h at 37°C) in the presence or absence of bafilomycin (10 nM). **b**) Numbers of toxin-induced vacuolated cells in the presence of varying concentrations of bafilomycin were counted and expressed as percentage of toxin-untreated control cells. All values are the mean of two different experiments run in triplicate ± SD.

Figure S2

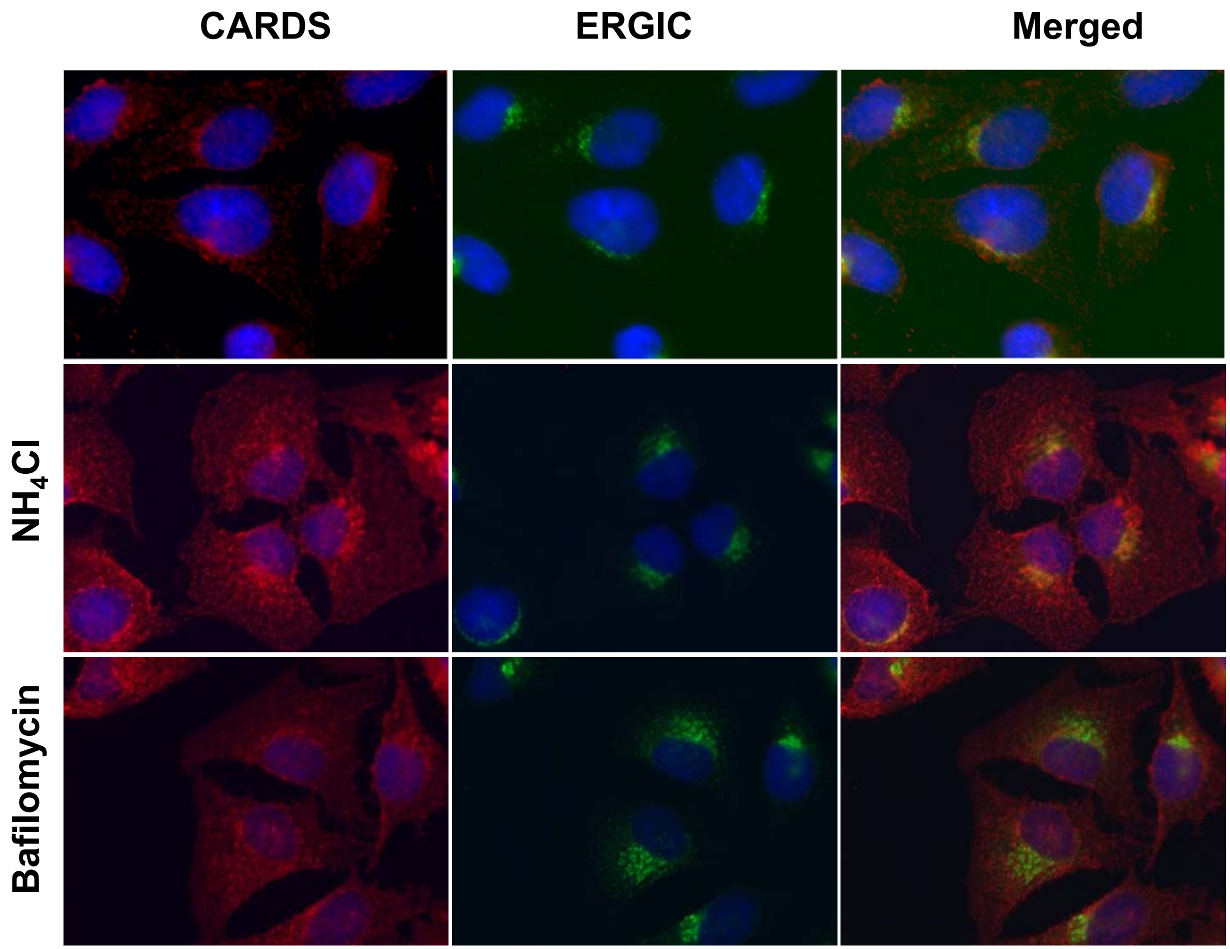

Figure S2. Effect of NH_4Cl and bafilomycin on CARDS toxin ERGIC transport. HeLa cells pretreated with and without NH_4Cl (5 mM) or bafilomycin (10 nM) were incubated with CARDS toxin (140 pmol) at 4°C for 1 h. Unbound toxin was removed, and cells were shifted to 37°C, incubated for 8 h and fixed in the presence of DAPI to stain nuclei (blue). Fixed cells were probed with rabbit polyclonal anti-CARDS toxin antibodies followed by secondary goat anti-rabbit IgG conjugated with Alexa Fluor 555 (red) and anti-ERGIC mouse monoclonal antibodies followed by secondary antibodies conjugated with Alexa Fluor 488 (green). Merged image shows co-localization of CARDS toxin and ERGIC as yellow color. Note dissipation of CARDS toxin throughout cytosol and its varying degrees of association with ERGIC in the presence of pharmacological agents.

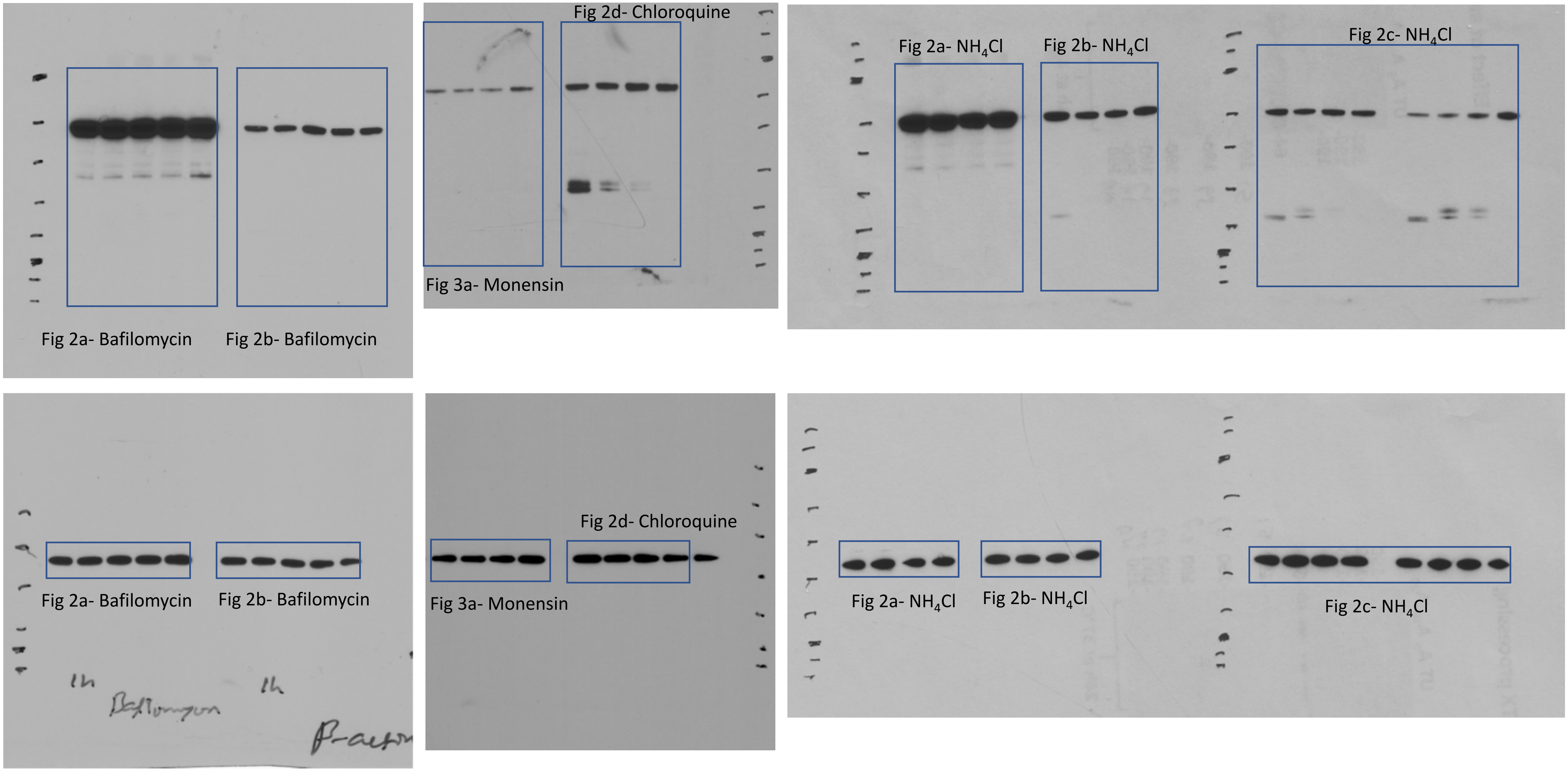
Fig 2a- Bafilomycin
Fig 2b- Bafilomycin
Fig 2d- Chloroquine
Fig 3a- Monensin
Fig 2a- NH_4Cl
Fig 2b- NH_4Cl
Fig 2c- NH_4Cl
Fig 2a- Bafilomycin
Fig 2b- Bafilomycin
Fig 2d- Chloroquine
Fig 3a- Monensin
Fig 2a- NH_4Cl
Fig 2b- NH_4Cl
Fig 2c- NH_4Cl

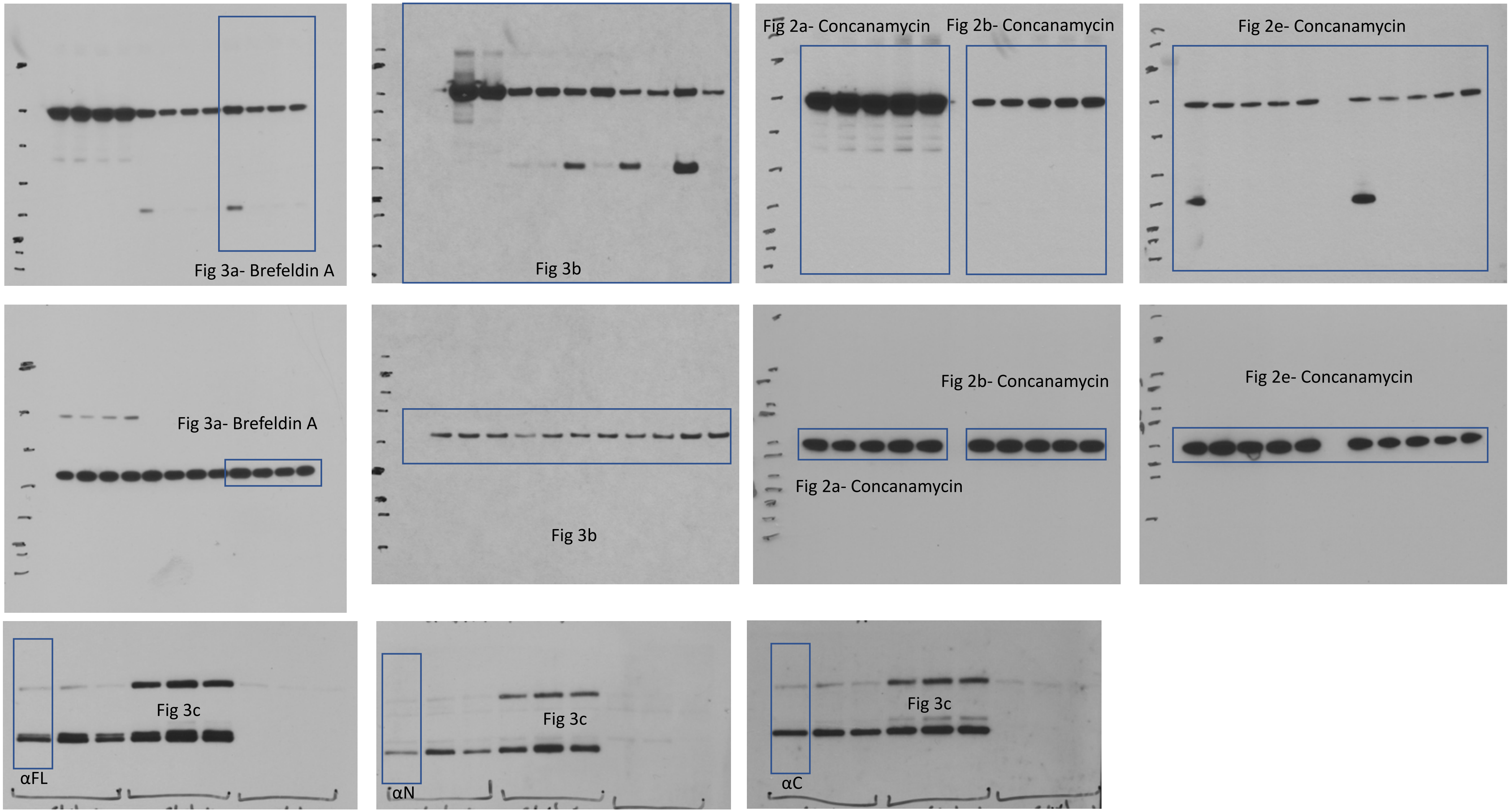

Fig 3a- Brefeldin A
Fig 3b
Fig 2a- Concanamycin
Fig 2b- Concanamycin
Fig 2e- Concanamycin
Fig 3a- Brefeldin A
Fig 3b
Fig 2b- Concanamycin
Fig 2a- Concanamycin
Fig 2e- Concanamycin
Fig 3c
αFL
Fig 3c
αN
Fig 3c
αC
